# Toward an Integrative Nursing Curriculum: Combining Team-Based and Problem-Based Learning with Emergency-Care Scenario Simulation

**DOI:** 10.3390/ijerph17124612

**Published:** 2020-06-26

**Authors:** Cheng-Yi Huang, Ya-huei Wang

**Affiliations:** 1 School of Nursing, Chung Shan Medical University, Taichung 40201, Taiwan; huangcy@csmu.edu.tw; 2 Department of Nursing, Chung Shan Medical University Hospital, Taichung 40201, Taiwan; 3 Department of Applied Foreign Languages, Chung Shan Medical University, Taichung 40201, Taiwan; 4 Department of Medical Education, Chung Shan Medical University Hospital, Taichung 40201, Taiwan

**Keywords:** team-based learning, problem-based learning, scenario simulation, core nursing competencies, emergency care

## Abstract

Objective: The study intended to combine team-oriented, problem-based learning (PBL) with emergency-care simulation to investigate whether an integrative intervention could positively impact the core nursing competencies and teacher performance of nursing students. Methods: The study belonged to the domain of action research, which aimed to address the weaknesses of traditional teacher-led, lecture-based learning. An 18-week, single-case experimental design, in which 58 senior nursing students at a medical university in central Taiwan participated, was conducted to test the possible benefits of the intervention. The measures included the Scale of Core Nursing Competencies and the Teacher Performance Evaluation Scale. Results: The research results showed that nursing students who received integrative training that combined team-based PBL with emergency-care scenario simulation had stronger mastery over core nursing competencies. At the same time, they also evaluated both the “Emergency Care” course for which the curriculum was used and the teachers’ performance in that course more highly. Conclusions: The findings suggest that an integrative curriculum combining team-based PBL with scenario simulation is worth pursuing. Compared with traditional teacher-led, lecture-based teaching, this curriculum may be more effective in helping nursing students develop core competencies in their field.

## 1. Introduction

Taiwan has long faced the challenges presented by a variety of natural and human-caused disasters. In these disasters, healthcare professionals including nurses have played an important role in providing immediate care and treatment, whether they are on call, working in hospital emergency rooms, or assigned to disaster sites where people have suffered critical illness or injuries. According to the report of the Ministry of Health and Welfare, each year there are nearly 3.8 million emergency patients in the Taiwanese healthcare system [1]. With higher rates of emergency department use, greater variability in patient diseases and injuries, and more uncertainty about the course of diseases, the Institute of Medicine (IOM) has estimated that every year there are as many as one million deaths or injuries caused by medical negligence or treatment errors [2].

Therefore, in order to be prepared to face the complexities and challenges of medical care, nurses should be trained to be collaborative, practice-ready professionals, capable of working in interprofessional teams in order to improve the quality of medical care, ensure patient safety, and, more generally, build a safe clinical environment. However, traditional teacher-led, lecture-based methods of instruction often fail to motivate nursing students being trained as healthcare providers to become educated in a way that will make them practice-ready.

In the clinical situation, emergency care is filled with high-stress, quickly changing scenarios; nurses and other healthcare providers are expected to have competencies involving not just their own professional skills, but also the capability of using their skills as part of larger medical teams [3]. For nursing professionals in particular, whether they are working in hospital emergency rooms or at disaster sites, there is an expectation that they will be proficient in core nursing competencies. Specifically, the expectation is that nurses will have the professional knowledge and skills needed to perform assessments of patients [4], while also being able to communicate with other team members to deal with various unexpected clinical issues such as chronic disease exacerbation or outbreaks of infectious diseases [5]. It is the combination of professional skills and collaborative teamwork that allows nurses, like other healthcare providers, to recognize, assess, and manage contingencies; help administer the proper kinds of care; and make appropriate ethical choices in the process [3,6].

In order to help students acquire these wide-ranging competencies, emergency-care education should extend beyond traditional teacher-centered, lecture-based teaching, also emphasizing “learning by doing” and the need for teamwork and collaboration. The same enriched curriculum should be offered to nursing students, who require a range of competencies in order to be able to manage possible problems and prepare for highly complex, quickly changing, and sometimes harsh medical-care environments. Problem-based learning (PBL), used worldwide in medical and nursing education [7], has been proven to be effective in education, with studies demonstrating that it can facilitate students’ self-directed learning and bring about positive learning outcomes such as self-learning ability, peer communication, teamwork, lifelong learning, problem solving, innovation, and collaboration [8,9,10]. Furthermore, compared with traditional classroom teaching methods, PBL can significantly improve the students’ professional communication skills [10]. However, in the past two decades, PBL has been applied to core courses in nursing such as pediatric nursing [11], fundamental nursing [12], medical-surgical nursing [13], and technical skills in nursing [14]. Most of these courses belong to the core courses and skills training that are part of traditional nursing curricula, rather than focusing on complex clinical tasks and collaborative problem-solving requiring teamwork. Moreover, as students in PBL classes are arranged in different groups and are spread across different PBL classrooms, students in different groups have no interaction with each other. Therefore, to retain the positive aspects of PBL while avoiding its limitations, team-based learning (TBL) has been used to facilitate inter-group communication in instructional environments.

Team-based learning (TBL) was initiated by Larry Michaelson, a management professor, in the 1970s, and later applied to medical education to facilitate theme-oriented discussions [15]. Generally, TBL is often conducted in a large class, with students divided into several groups and with one or two mentors serving as facilitators to guide the group-learning activities and thematic discussions [16]. With the assistance of TBL, via discussion of pre-planned themes and critical-thinking practice, students can reflect on the proposed problems through intra-team and inter-team cooperation and collaboration, thereby finding optimal solutions. In this way, TBL can overcome the limitations while still retaining the strengths of PBL, potentially making TBL a more efficient and cost-effective teaching strategy than PBL used alone [16]. As with PBL, TBL allows self-directed, experiential learning; however, it can further facilitate interconnected teamwork and collaborative forms of practice [16,17].

As patient safety is the primary concern, it is too risky to carry out experiential-learning activities through clinical practice at real clinical sites. Research has shown that 54% of medical errors including errors in the emergency-care process is due to nurses [18,19]. Novice nurses, lacking clinical experience and critical-thinking skills, are especially error-prone [20]. To prevent such medical errors in a clinical setting, the most effective strategy is to incorporate situational, scenario-driven training exercises into nursing education and professional training. These exercises can help students acquire basic and advanced nursing knowledge and skills [21]. Scenario-simulation teaching has proven effective in medical and nursing education. Kenaszchuk et al. [22], for example, applied scenario simulation to collaborative teamwork and training, with a view to facilitating professional communication among different disciplines and thereby reducing the risk of medical errors. Zarifsanaiey, Amini, and Saadat [23] used simulation-based training as a clinical teaching strategy in nursing education to improve the students’ nursing performance levels and critical-thinking competence. As scenarios simulating real-world clinical situations can provide a safe environment for the repetitive practice of nursing skills, these have played an important role in emergency care and critical medical care [24]. The use of such scenario simulations has improved inter-professional communication among members of medical teams, enhanced team efficiency, facilitated interpersonal understanding and cooperation, and increased the team members’ overall professional competence when dealing with complex medical problems [25].

The creation of clinical-situation scenarios can help facilitate the integration of nursing knowledge, skills, and training, enabling nursing students to reach key learning goals with respect to cognition, skills, and care provision [26]. All nursing schools offer emergency-care courses for senior nursing students to help them acquire the knowledge and other forms of competence required for emergency care. However, the instructional strategies used in such emergency-care courses have focused almost exclusively on the transmission of content. These pedagogical strategies can no longer meet the requirements of the 21st century, with its many technical challenges and rapidly shifting healthcare needs. Already faced with the challenges presented by high-tech medical equipment and quickly evolving diseases, nursing professionals must also be skilled at teamwork, demonstrating collaborative competence, and cross-disciplinary communication skills, if they are to be able to provide professional nursing care [27].

In an effort to enhance nursing students’ capacity for collaborative problem solving and professional nursing skills, the present study aimed to use an integrative curriculum that combined team-based PBL with emergency-care scenario simulations to enable students to share and thereby enhance their knowledge and skills through team discussions. The study explored whether an integrative intervention of this sort might have a positive impact on the nursing students’ core competencies. By integrating scenario simulations with team-based PBL, the goal was to solidify their nursing knowledge and skills to strengthen their core nursing competencies as well as their competence in team-based collaborative care, thereby providing better nursing care to those in need. The study also explored whether this curricular intervention might also improve the teachers’ performance.

## 2. Material and Methods

In order to investigate the feasibility of using an integrative intervention to increase the nursing students’ core nursing competencies and teacher performance, the overall research objectives of the study were as follows:

➢ To compare how two different groups of students, namely, students exposed to the integrative curriculum vs. students exposed to traditional teaching methods, performed on tests designed to gauge their core nursing competencies.
➢ To compare the students’ actual performance on tests of their core nursing competencies with the performance that instructors expected from them.
➢ To compare how the two different groups of students, namely, students exposed to the integrative curriculum vs. students exposed to traditional teaching methods evaluated their teacher’s performance.

### 2.1. Participants and Research Design

The study exemplified action research, which focused on real-world situations with the aim of helping participants in those situations develop the knowledge and competencies required to (re)evaluate and improve them [28,29]. In order to address the weaknesses of traditional teacher-led, lecture-based classroom environments, the study used simulated scenarios to inspire the students’ active learning. A single-case experimental design was employed to test the possibility of using team-based PBL combined with emergency-care scenario simulations to increase the performance of nursing students in their core competency areas as well as the performance of the instructors teaching the integrative course. The emergency care course was an elective course for senior nursing students. The research objectives, design, strategies, and measurements were launched on the website one month before the research was to take place, in order to let students have a full understanding of the research framework before being willing to participate in the course. In addition, for those enrolling in the course, they had to provide consent for participating in the course via the school informatics system. The students participating in the course were the senior students at a medical university in central Taiwan, with an age range between 22 and 24. There were 58 participants exposed to the integrative curriculum, in which there were 47 (81.03%) female students and 11 (18.97%) male students. The baseline used to compare the students exposed to the integrative curriculum were the students exposed to the traditional curriculum. There were 51 students exposed to the traditional curriculum, in which there were 41 (75.61%) female students and 10 (24.39%) male students. All of the students had finished their study of core nursing courses. The two-credit “Emergency Care” ran for 18 weeks, with two sessions per week from September 2015 to January 2016. However, during the 18-week intervention, the students situated in the integrative curriculum were required to keep “reflection logs,” in which they recorded comments about the proposed themes related to emergency care.

The instructors of the course each had 12 years of experience in teaching this class. In addition, before becoming a teacher at a medical university, the lead instructor had extensive clinical nursing experience in front-line emergency care. Hence, the lead instructor had a deep understanding of various emergency-care situations, the demands on nurses in those situations, and the roles that nurses are expected to play at clinical sites. Moreover, all of the instructors who participated in the course had received advanced training as PBL seed teachers, and had also participated in PBL teaching for many years in their respective disciplines.

### 2.2. Toward an Integrative Curriculum: Combining Team-Based Problem-Based Learning (PBL) with Emergency-Care Scenario Simulation

For this study, in an effort to help nursing students increase their core nursing competencies, in the Emergency Care course, instead of using the traditional teacher-led, lecture-based learning, the instructors used the integrative approach described previously. The research assumption was that this integrative curriculum, by combining team-based PBL with scenario simulations, could help nursing students achieve stronger mastery over core nursing competencies (compared with the abilities achieved via traditional classroom instruction). The integrative curriculum was designed by the instructors after a series of discussions about what competencies needed to be targeted, what learning goals should be set, what teaching schedule should be followed in the Emergency Care course, and so on. Once the curriculum was drafted, it was sent to two external curriculum reviewers, who were also nursing professionals, who evaluated the content. The curriculum reviewers confirmed the course’s content validity and found the proposed curriculum to be appropriately designed; they also found that the emergency-care scenario cases proposed for the course were in line with clinical practice. Furthermore, in order to create the scenario simulation, in the simulation room used at the teaching site, there were simulated patients whose changes in blood pressure, pulse, respiration, blood oxygen saturation concentration, and electrocardiogram results the nursing students had to monitor, so as to practice providing on-time nursing care and case management.

The framework for the course and the instructional procedures that were used to teach it, along with a sample from the teaching manual, are shown in Table 1, Table 2 and Table 3, respectively.

### 2.3. Teaching Procedure

The Emergency Care course was an 18-week course covering everything from the causes of injuries to the development of emergency-care competencies. In the intervention, the researchers used the integrative curriculum to facilitate both scenario-simulation teaching and the team-based PBL discussion. To facilitate the team-based PBL discussion for the scenario cases, teaching/learning goals were divided into three stages (see Table 2). The first stage focused on defining the patients’ healthcare problems and needs to form possible assumptions about the problems in question. In this stage, the instructors guided the nursing students to apply their prior knowledge to identify possible mechanisms for these assumed problems. The second stage focused on defining the learning issues for students, the kinds of self-study (i.e., self-directed inquiry) needed to address those issues, and the application of nursing skills to the problems at hand. The third stage focused on the interaction of new knowledge and skills with previously established competencies to solve case problems. At this stage, the instructors provided feedback and evaluation to the students for further revision of their approaches to the case problems. This teaching method is shown in Table 2.

Before proceeding to the team-based PBL case discussion, the students were arranged in learning teams, with each team consisting of 9–10 students. There were therefore six teams in total. As the integrative curriculum was designed to improve the nursing students’ core competencies, the course took into account the ranking of the top 10 causes of death and accident injuries in Taiwan. More specifically, the instructors selected relevant scenario cases, chose thematic issues, and designed lesson plans around highly ranked causes of death and injury. The following sample from the course teaching manual (Table 3) presents a scenario case about a patient with acute coronary syndrome.

### 2.4. Measures

#### 2.4.1. Scale of Core Nursing Competencies

After the intervention via the integrative curriculum, these nursing students were required to log into the school’s learning website to anonymously fill in a scaled questionnaire about the core nursing competencies, which was developed by the nursing teachers at the medical university the researchers work at. There were six core competencies in the scale: (1) professional nursing knowledge and skills, (2) critical thinking and problem-solving abilities, (3) lifelong learning ability, (4) professional identity and commitment, (5) maturity and capacity for collaboration and teamwork, and (6) respect for lives and ethics. The scale was set up as a five-point Likert scale, from 5 (totally agree) to 1 (totally disagree). The higher the score, the higher the (self-rated) improvement of the competence. The Cronbach’s alpha for the scale was 0.78.

#### 2.4.2. Teacher Performance Evaluation Scale

A teacher performance evaluation scale was used to gather the students’ feedback on the instructors’ teaching. The evaluation scale was a standardized scale developed by the medical university the researchers work for. Examples of items are: “Teaching material and curriculum help improve the learning effect” and “The instruction is clear and vivid, helping enhance students’ learning motivation.” Students once again had to log in to the school’s website to fill in the scaled questionnaire anonymously. The scale was arranged as a five-point Likert scale, with 5 meaning “strongly agree” and 1 meaning “strongly disagree.” It covered five dimensions including “teaching material and curriculum”, “teaching method and instruction”, “extra-curricular tutoring”, “assessment”, and “teacher-student interaction”. The higher the score, the more favorable the students’ evaluation of the course and the teachers. The Cronbach’s alpha for the scale was 0.92.

#### 2.4.3. Students’ Reflection Logs

In order to examine the feasibility of the integrative curriculum, this study used the students’ reflection logs as a qualitative instrument to triangulate the scale results. Additionally, through the analysis of the students’ reflection logs, the instructors could identify the strengths and weaknesses of the intervention, allowing them to improve future iterations of the course.

### 2.5. Data Analysis

**Figure 1:**
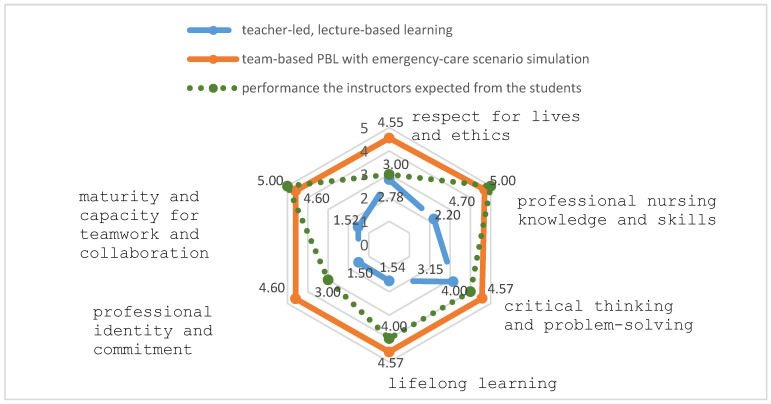
A comparison of the nursing students’ performance in six core competencies.

In order to determine the impact of the intervention, the study compared the performance of the students exposed to the integrative curriculum with the performance of the students exposed to the traditional curriculum. The radar chart in Figure 1 shows the results of this comparison. The chart also shows the difference between the actual performance of the students against their expected performance, with respect to the acquisition of core nursing competencies. The results of the teacher performance evaluation, meanwhile, were presented in an XY scatter diagram. The data analysis method used here was the TOPSIS (Technique for Order Preference by Similarity to Ideal Solution) method [30] to further understand the students’ overall evaluation and dimensional evaluations of the course and the teachers’ performance. Finally, in order to gain a holistic understanding of the students’ reaction to the intervention, the researchers analyzed the qualitative feedback from the reflection logs and triangulated that data with the quantitative results.

## 3. Results

To compare the performance of the two groups of nursing students in core nursing competencies (i.e., the performance of the group exposed to the integrative curriculum and the group exposed to the traditional curriculum), a radar chart was developed (see Figure 1). As shown in Figure 1, compared to those not receiving the intervention in their final academic year (means = 2.20, 3.15, 1.54, 1.50, 1.52, and 2.78), the students exposed to the integrative curriculum felt that after the intervention, they had higher core nursing competencies (means = 4.70, 4.57, 4.57, 4.60, 4.60, and 4.55) in professional nursing knowledge and skills, critical thinking and problem-solving abilities, lifelong learning ability, professional identity and commitment, maturity and capacity for teamwork and collaboration, and respect for lives and ethics. In other words, they used the self-ratings to indicate strong improvements in their core competencies in the field.

Moreover, the radar chart also shows the differences between the students’ self-ratings and the performance level the instructors expected from the students. In the areas of critical thinking and problem-solving, lifelong learning, professional identity and commitment, and respect for lives and ethics, the students’ self-ratings (means = 4.57, 4.57, 4.60, and 4.55) were higher than the performance level the instructors expected from the students (means = 4.00, 4.00, 3.00, and 3.00). In the areas of “professional nursing knowledge and skills” and “maturity and capacity for collaboration and teamwork”, the instructors’ expected levels (means = 5.00 and 5.00) were higher than the students’ self-assigned values (means = 4.70 and 4.60).

**Figure 2:**
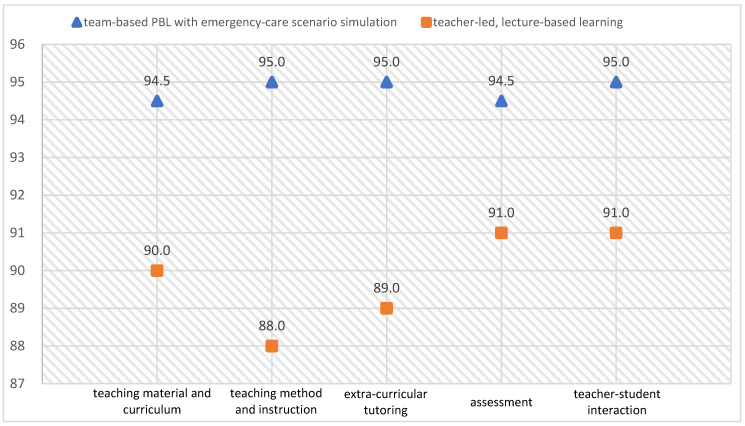
A comparison of the students’ course and teacher evaluations.

The researchers also compared the two groups of student evaluations of the course and the teachers’ performance. As shown in Figure 2, an XY scatter diagram, compared to the group not receiving the intervention in their final academic year (means = 90.0, 88.0, 89.0, 91.0, and 91.0), the students exposed to the integrative curriculum rated both the Emergency Care course and the instructors’ performance more highly (means = 94.5, 95.0, 95.0, 94.5, and 95.0) along the dimensions of “teaching material and curriculum”, “teaching method and instruction”, “extra-curricular tutoring”, “assessment”, and “teacher-student interaction”.

The study further used TOPSIS [30] to analyze the overall evaluation and dimensional evaluations of the course and the teachers’ performance for the students exposed to the integrative curriculum and the students exposed to traditional teaching methods. There are seven steps used in the TOPSIS method, shown as below:

Step 1. To establish the decision matrix (*D*), which consists of the alternatives (*A_i_* for *I* = 1, 2,…, *m*), criteria (*C_j_* for *j* = 1, 2,…, *n*), and student course and teacher evaluations (*x_ij_*). Equation (1) shows the decision matrix (D).

(1) $$D=\begin{array}{cc} & \begin{array}{ccc} C_{1} & \ldots & C_{n} \end{array}\\ \begin{array}{c} A_{1}\\ \ldots \\ A_{m} \end{array} & \left(\begin{array}{ccc} x_{11} & \cdots & x_{1n}\\ \vdots & \ddots & \vdots \\ x_{m1} & \cdots & x_{mn} \end{array}\right) \end{array}$$

Here in the study, the alternatives were the integrative curriculum and traditional teaching methods. The criteria included “teaching material and curriculum”, “teaching method and instruction”, “extra-curricular tutoring”, “assessment”, and “teacher–student interaction”. Hence, the following decision matrix was established and shown in Table 4.

Step 2. To normalize *x_ij_* in order to allow the comparison of the various criteria. The normalize value *r_ij_* is calculated as Equation (2).

(2) $$r_{ij}=\frac{x_{ij}}{\sqrt{\sum_{i=1}^{m}x_{ij}^{2}}}$$

Step 3. To establish the weighted normalized decision matrix. The weighted normalized value *p_ij_* was calculated as Equation (3). *w_i_* is the weight for each criteria. The weight value was set at 0.2 for each criteria.

(3) $$p_{ij}=w_{i}*r_{ij}$$

Step 4. To define the positive ideal solutions (*P*^+^) and negative ideal solutions (*P*^−^). *P* ^+^ is obtained by $\left[\underset{j}{\max}p_{ij}\right]$ and P^−^ is obtained by $\left[\underset{j}{\min}p_{ij}\right]$.

Step 5. To calculate the Eulidean distances for the *S*^+^ and *S*^−^. The Eulidean distance for the *S*^+^ is described in Equation (4) and *S*^−^ is as per Equation (5).

(4) $$S_{i}^{+}=\sqrt{\overset{n}{\underset{j=1}{\sum }}{\left(p_{ij}-p_{j}^{+}\right)}^{2}}$$

(5) $$S_{i}^{+}=\sqrt{\overset{n}{\underset{j=1}{\sum }}{\left(p_{ij}-p_{j}^{+}\right)}^{2}}$$

Step 6. To calculate the relative closeness coefficient *C_i_* for each alternative concerning the positive ideal solution. Equation (6) shows the formulation *C_i_*.

(6) $$C_{i}=\frac{S_{i}^{-}}{S_{i}^{+}+S_{i}^{-}}$$

Step 7. To rank the alternatives according to the relative closeness coefficient.

The higher the *C_i_* value, the better the alternative. That is, the alternative with higher *C_i_* value is closer to the positive ideal solution. Here, *C_2_* is 1 and *C_1_* is 0. Hence, the overall performance showed that the integrative curriculum (*C_2_*) had an obviously higher rank than that of the traditional teaching methods (*C_1_*). The calculation process and the derived results from Steps 1 to 7 are shown in Table A1, Table A2, Table A3 and Table A4.

In addition, to gain a more holistic understanding of the student reactions to the integrative curriculum, the researchers summarized the data from the students’ reflection logs as follows. The students stated that the instructors in the integrative course, instead of directly providing all the answers about the problem cases, led a discussion that promoted self-directed inquiry by giving clues and key points to help students engage in team-based discussions about the scenario cases. Hence, the students could independently reflect on the cases critically and collaboratively settle on a solution to the problems. Moreover, while engaged in these team-based discussions, the students had more interaction with their peers as well as their instructors, collaboratively figuring out the mechanisms behind the problem cases, and in this way acquiring more of the professional nursing knowledge and skills needed to solve the problems.

In addition, students reflected that while participating in the emergency-care simulation scenarios, they found themselves more enthusiastic about the course: they were excited about encountering the different scenarios. The students also felt that the use of team-based PBL combined with scenario simulation enriched the class instruction and activities, and that it gave them more opportunities to practice applying their professional knowledge and skills in the clinical cases. Hence, through more demonstration of and practice with the targeted use of nursing skills, students felt that they better acquired core competencies in the field.

## 4. Discussion

The study aimed to use team-based PBL combined with emergency-care scenario simulations to enable students to share and thereby enhance their knowledge and skills through team discussions. The research assumption was that, through collaboration in the simulated scenarios, nursing students could sharpen their professional knowledge and competencies, learning how to reflect on and find optimal solutions for the scenario case problems. In addition, through participation in the scenarios, students could learn how to be committed members of the nursing field by maintaining a positive professional identity and attitude and respecting others.

The research results demonstrate that the use of the integrative curriculum can have a positive impact on student outcomes as well as teacher performance. After participating in the curriculum, students rated more highly their own professional nursing knowledge and skills, critical thinking and problem-solving abilities, lifelong learning ability, professional identity and commitment, maturity and capacity for collaboration and teamwork, and respect for lives and ethics. The results confirm Burgess et al.’s [16] finding that team-based learning combined with a PBL medical curriculum can motivate students to learn more independently and to support each other through team discussions. In addition, students become more capable of recognizing problems and enable themselves to seek appropriate solutions for those problems [7].

Moreover, in order to solve the problem cases used in the simulated scenarios, students were more inclined to collaborate and communicate with their fellow team members in order to address the medical issues involved. The research results confirm Shin, Park, and Kim’s [31] finding that scenario-simulation teaching can enhance the students’ motivation to learn as well as their attitudes about learning. The students’ learning performance in the core nursing competencies also corresponded with Kong et al.’s [9], Li, Wang, Zhu, Zhu, and Sun’s [10], and Wosinski et al.’s [32] findings that PBL teaching can have positive impacts on students’ problem-solving abilities, and capacity for teamwork and collaboration. Additionally, the use of simulation-based training as a clinical teaching strategy in nursing education can improve the students’ nursing performance levels and critical-thinking competence [23,32].

The research results also demonstrate that the integration of the scenario simulation with team-based PBL foregrounds inter-group and intra-group interactions, hence facilitating learning and increasing students’ motivation to learn. Though PBL has been demonstrated to be useful in facilitating student learning performance such as self-directed learning and critical thinking [8,9], while being arranged in different groups and being spread across different PBL classrooms, students have no interactions with other group members. However, the integration of the scenario simulation with team-based PBL can address the weakness of PBL practice, in that the students exposed to the integrated curriculum are divided into several groups and stay in the same classroom, with mentors serving as facilitators to guide the group-learning activities and thematic discussions [16]. Therefore, these students can have more experience of presenting their ideas in a scenario to facilitate both inter-group and intra-group interactions. Moreover, the use of scenario simulation in the integrative curriculum has also improved the students’ teamwork and collaboration. The research results also correspond with Norman’s [25] study in that the use of scenario simulation can facilitate interpersonal cooperation and team efficiency, and hence increase the students’ core nursing competencies.

The positive improvements in the core competencies also induced the students to rate the course and the instructors favorably. As the integrative curriculum fosters a learner-centered class environment in which the instructors mainly serve as facilitators who give on-time assistance to the participants, the students themselves have to engage with the case problems and collaboratively solve them in the simulated clinical setting. To this end, students have to incorporate their prior knowledge into the simulation scenarios and use it as a basis for collaboration and teamwork. In this way, they can acquire, through team discussions, new knowledge that builds on their prior knowledge. The research results thus support Darling-Hammond et al.’s [33] finding that successful teaching depends on enabling students to use their prior knowledge in classroom contexts. In the study, the researchers successfully created simulation scenarios where students could naturally and organically learn from and with their teammates, using their previous knowledge. The students were thus better positioned to become self-directed in developing their professional knowledge and skills; in other words, the course made them autonomous and independent learners. The significance of the study involves an integration of the scenario simulation with team-based PBL to address the weakness of PBL practice, that is, to retain the positive aspects of PBL while avoiding its limitations. Moreover, the integrative curriculum combining team-based PBL with scenario simulation can facilitate both intra-group and inter-group communication to help students acquire the core nursing competencies necessary for dealing with complex medical problems in real clinical situations. It is thus no wonder that these students were satisfied with the course as well as the teachers’ performance.

## 5. Conclusions

The study successfully demonstrated the feasibility of using the integrative curriculum to enable students to be the center of their own learning process and to draw on their prior knowledge to manage case problems in the simulated clinical scenarios. The findings also suggest that an integrative curriculum combining team-based PBL with scenario simulations is worth pursuing. Compared with traditional teacher-led, lecture-based teaching, this curriculum may be more effective in helping nursing students develop core competencies in their field. The study belongs to the domain of action research, which aims to address the weaknesses of traditional teacher-led, lecture-based learning, in this case, by using simulated scenarios to inspire the students’ active learning. The main limitation is that the students in the research could not be randomly selected. In future studies, researchers may wish to employ a quasi-experimental design or a true experimental design to further verify the feasibility of using the integrative curriculum for nursing instruction. Those interested in the study may also wish to test the intervention in different simulated clinical settings such as hospice palliative care, intensive care, or elderly care.

## Figures and Tables

**Table 1:** Framework for the integrative curriculum (team-based PBL combined with emergency-care scenario simulation).

Stage	Task
Analysis & Design	Integrating social and healthcare needs, identifying causes of accidents and injuries, and defining the goals of the various nursing disciplines
	Defining instructional themes and designing specific lesson plans
Development	Developing lesson plans, setting the learning goals for each emergency-care scenario, and formulating the scenario content
	Rechecking/confirming the teaching and learning strategies to be used in the course including the particular steps that will be followed to integrate team-based PBL with emergency-care scenario simulations
	Confirming the evaluation tools that will be used to measure student outcomes
Implementation & Application	Team arrangement and task assignment
	The team-based PBL case discussion
Evaluation	Evaluating learning outcomes
	Understanding the reactions of students and instructors
	Improving and revising the lesson plans

**Table 2:** Teaching procedure used for the integrative curriculum.

Stage	Step	PBL Themes	Teaching Methods	Teaching Content
I	1	Defining healthcare problems and needs (time: 10 min)	Lecture and instruction Team-based discussion	Providing emergency-care scenario cases for the team-based PBL Collaborative study of the emergency-care scenario cases to identify crucial facts and clues, with the aim of determining what kinds of care should be provided.
	2	Exploring the areas of knowledge students have mastered previously (time: 20 min)	Team-based discussion	Applying past pathophysiology knowledge and nursing care skills to explore the possible health needs in the emergency-care scenario cases
	3	Forming assumptions and identifying possible mechanisms (time: 15 min)	Team inquiry Instructor-guided discussion of assumptions and mechanisms	Applying prior knowledge and skills to establish causal relationships, potential biopathological mechanisms, and relevant assumptions Developing nursing care options Reading the prepared reference material to think critically about the feasibility of implementing nursing care
II	4	Defining learning issues (time: 10 min)	Team-based discussion Logical induction	Setting learning goals Logical induction after team-based discussion to confirm primary and secondary learning goals Constructing a conceptual framework based on learning content
	5	Self-study & application of nursing skills (time: 45 min)	Self-learning, i.e., self-directed inquiry Demonstration and practice	Providing learning resources Students acquire, analyze, and apply new knowledge and skills in order to develop possible nursing-care options Nursing-care skills demonstration and practice
III	6	The integration of new knowledge and skills with prior knowledge to solve case problems (time: 50 min)	Scenario simulation Skills practice and demonstration	Class presentations Simulating situational communication and collaboration to solve the healthcare problems in the scenario cases Hands-on skills practice and demonstration
	7	Evaluation and reflection (time: 50 min)	Team observation, discussion, and reflection	Feedback and evaluation Knowledge and skills revision

**Table 3:** Example from the teaching manual: a scenario involving a patient with acute coronary vascular syndrome.

Teaching Manual	Content
Scenario Case	Topic: A middle-aged father with severe chest pain
Preface	Students’ prerequisite knowledge: basic medical science, medical-surgical nursing
	Learning goals: developing and integrating nursing students’ competencies in cardiovascular medicine and nursing knowledge, emergency-care skills, communication, and teamwork and collaboration
	Lesson plan summary: briefly explaining the content of the lesson plan and the theme to be explored
	Class management: the distribution of scenario case material every week
Scenario Content	Mr. Chang, 50 years old, comes to the emergency department for medical treatment because of his shortness of breath and his difficulties walking. Over the past week, his feet have been swollen, his weight has increased by 2 kg, and his whole body has felt weak and tired. In the middle of the night, he often wakes up with difficulty breathing. Today, just by moving around, he once again feels shortness of breath and breaks into a cold sweat. He therefore goes to an emergency department for treatment. The doctor’s diagnosis is CHF FC: III/IV. You are an emergency nurse; please show us how you will carry out your nursing assessment.
	Main background information:
	Medical history: The patient has had a history of hypertension for the past 5 years, but he has not been taking medication according to the doctor’s orders. After taking a short rest, he usually recovers enough to catch his breath and continue with his activities.
	Emergency vital signs: TPR: 36 °C, 90 times/min, 26 times/min, BP: 160/90 mmHg. SpO2: 93%.
	Evaluation data from a systematic physical examination:
	Respiratory system: auscultation of lung-breath sounds reveals rales in the lower lobes on of the lungs
Teaching Guide	Key learning points in the scenario simulation case:
	Systematically collect information relating to this patient’s circulatory problems—both subjective and objective information
	Common nursing problems for emergency patients with circulatory disturbance
	Emergency nursing management of patients with decreased cardiac output
Key Points	Decreased cardiac output
	CHF (congestive heart failure)
Learning Issues	Introducing the key points that students must discuss in this scenario-simulation case:
	Myocardial tissue perfusion system
	Emergency treatment and management of decreased cardiac output
	Definition of heart failure and its pathophysiology and treatment/management
Raising Questions	Use the key points of study to write out brief questions for which the instructors’ guidance is sought
	Example: How do I help the patient get relief from the symptoms of dyspnea, such as gasping for breath?
References	Brief reference materials provided by the instructors:
	Pathophysiological signs of patients with heart failure
	Emergency care for patients with heart failure

**Table 4:** The decision matrix for the technique for order preference by similarity to ideal solution (TOPSIS).

	Criterion				
Alternative	Teaching Material and Curriculum	Teaching Method and Instruction	Extra-Curricular Tutoring	Assessment	Teacher-Student Interaction
Traditional Teaching Methods	90	88	89	91	91
Integrative Curriculum	94.5	95	95	94.5	95

## Appendix A. The Calculation Results for TOPSIS

**Table A1:** The normalized decision matrix.

	Criterion				
Alternative	Teaching Material and Curriculum	Teaching Method and Instruction	Extra-Curricular Tutoring	Assessment	Teacher–Student Interaction
Traditional Teaching Methods	0.689655	0.679562	0.683686	0.693642	0.69174
Integrative Curriculum	0.724138	0.733618	0.729777	0.72032	0.722146

**Table A2:** The weighted normalized decision matrix.

	Criterion				
Alternative	Teaching Material and Curriculum	Teaching Method and Instruction	Extra-Curricular Tutoring	Assessment	Teacher–Student Interaction
Traditional Teaching Methods	0.137931	0.135912	0.136737	0.138728	0.138348
Integrative Curriculum	0.144828	0.146724	0.145955	0.144064	0.144429

**Table A3:** The positive ideal solutions (*P* ^+^) and negative ideal solutions (*P ^−^*).

	Criterion				
Ideal Solution	Teaching Material and Curriculum	Teaching Method and Instruction	Extra-Curricular Tutoring	Assessment	Teacher–Student Interaction
*P* ^+^	0.144828	0.146724	0.145955	0.144064	0.144429
*P* ^−^	0.137931	0.135912	0.136737	0.138728	0.138348

**Table A4:** The Eulidean distance, the relative closeness coefficient, and rank.

Alternative	*S* ^+^	*S* ^−^	*C_i_*	Rank
Traditional Teaching Methods	0.017745	0	0	2
Integrative Curriculum	0	0.017745	1	1

*S*^+^—the target alternative to the best condition; *S*^−^—the target alternative to the worst condition.
